# Should Gender Reassignment Surgery be Publicly Funded?

**DOI:** 10.1007/s11673-018-9881-6

**Published:** 2018-11-05

**Authors:** Johann J. Go

**Affiliations:** Worcester College, University of Oxford, Walton Street, Oxford, Oxfordshire, OX1 2HB UK

**Keywords:** Transgender, Gender reassignment surgery, Health resource allocation, Distributive justice

## Abstract

Transgender people have among the highest rates of suicide attempts of any group in society, driven strongly by the perception that they do not belong in the sex of their physical body. Gender reassignment surgery (GRS) is a procedure that can change the transgender person’s physical body to accord with their gender identity. The procedure raises important ethical and distributive justice concerns, given the controversy of whether it is a cosmetic or medical procedure and the economic costs associated with performing the procedure. This paper argues that there is a strong case for funding GRS as a matter of clinical necessity and justice. This paper will be divided in four key sections: First, the state of transgender health will be outlined, including the role of GRS and common objections to it. Second, a number of common objections to GRS will be analysed at the outset and shown to be unconvincing. Third, a constructive argument will be advanced, arguing that publicly funded GRS is clinically necessary, cost-effective, and demanded by principles of justice. Fourth, the paper will briefly discuss moralistic biases and why we demand a higher burden of justification for funding GRS compared with other analogous procedures.

## Introduction

Healthcare rationing is inevitable. There are finite health resources for an almost infinite number of health needs. Given this reality, this paper analyses whether gender reassignment surgery (GRS) should be funded using our finite health budget and, if so, on what grounds. The issue of publicly funding gender reassignment surgery is fraught with immense difficulty, with complex ethical issues arising from clinical, policy, and economic considerations. The purpose of this paper is to argue that healthcare systems should publicly fund GRS and, where it is already funded, should make it more accessible to patients. The paper serves as additional affirmation for those jurisdictions who already fund GRS, showing that their policies are in line with their ethical and clinical obligations. Transgender persons are those whose physical or assigned sex does not accord with their gender identity (American Psychiatric Association [APA] 2013). According to the Diagnostic and Statistical Manual of Mental Disorders (DSM), transgender persons generally suffer from gender dysphoria (GD), which is the clinical distress associated with not fitting in their physical sex (APA 2013). I will hereinafter use the terms GD and transgender interchangeably. Across virtually all measures of physical and mental health, transgender persons have poorer outcomes than their non-transgender counterparts (Reisner et al. 2016). Compared to non-transgender people, transgender persons have higher rates of drug and alcohol abuse, HIV seroprevalence, diabetes, suicide ideation, and suicide attempts (Reisner et al. 2016). There is evidence to suggest that as many as 50 per cent of transgender youth experience suicide ideation and as many as 32 per cent have attempted suicide (Clements-Nolle, Marx, and Katz 2006; Grossman and D‘Augelli 2011). The primary contributor to these poor health outcomes is the transgender person’s strong psychological dissatisfaction with the fact that their physical sex does not correspond to their gender identity (Grossman and D‘Augelli 2011).

Gender reassignment surgery is promoted by the world’s leading medical authority on the issue, the World Professional Association for Transgender Health (WPATH), as an effective potential treatment for those whose GD meet specific clinical criteria (WPATH 2011). The clinical rationale for GRS is to alleviate the severe psychological angst the transgender person experiences as a result of their gender identity not aligning with their physical sex. Gender reassignment surgery can reduce or eliminate the psychological distress and is strongly associated with the prevention of suicide which might otherwise be attempted (Clements-Nolle et al. 2006; Grossman and D’Augelli 2011). It is currently offered in the United Kingdom in a limited capacity, with 457 operations performed in the last financial year (NHS, e-mail message to author, May 22, 2018).^1^ In New Zealand, a very small number of operations are offered each year subject to very strict conditions, though the waiting list is significant due to a lack of willing surgeons to perform the procedure (Ministry of Health 2012, 2017).

## The Problem of GRS Funding

The philosophical literature on GRS is extremely limited, with scant publications focusing on the ethics of publicly funding the procedure. While a range of ethical issues surround the funding of GRS, space constraints necessitate the setting of some parameters for this discussion. First, this paper is concerned only with the funding of gender reassignment surgery in jurisdictions with a state-funded universal healthcare system. It is not concerned with the issue of individual patients having the right to access privately funded GRS. Second, I will assume that those seeking GRS are of legal adult age, competent, and seeking the treatment voluntarily. Third, I will not undertake an analysis of whether or not GD should even be classified as a health issue or not. Arguments in other fields such as sociology have sought to remove GD as a clinical pathology and to instead treat it as a variation of the norm (Ault and Bryzuzy 2009). This issue is beyond the scope of this paper, but it should be noted that if GD is removed as a diagnosable clinical condition, it may have implications for transgender persons’ *health-based* claim to GRS and may therefore affect their ability to draw on the arguments I intend to present. I will instead take the approach of the DSM-5, which classifies GD as a diagnosable mental health condition.

The proposal for publicly funding GRS is, not surprisingly, often met with controversy and strident objections. There are three primary objections to publicly funding GRS: First, GRS may be opposed on the grounds that it is supposedly a cosmetic or enhancement procedure rather than a medical one (NHS 2018). Second, those who oppose GRS may advance the claim that it is not cost-effective and that the conditions of scarcity and opportunity costs do not support its funding. Third, a slippery slope argument may be advanced to oppose the public funding of GRS. This argument suggests that if we fund GRS, we will inexorably have to fund other procedures such as elective cosmetic surgery or race-alteration surgery. I demonstrate that these arguments are faulty and unconvincing.

The first objection is what we may call the cosmetic objection. This objection argues that GRS is a cosmetic procedure rather than a clinically necessary one and that we should therefore not fund it. Consider the basic argument structure below:

- P1GRS is a cosmetic procedure.
- P2The state should not publicly fund cosmetic procedures.
- CTherefore, the state should not publicly fund GRS.

The problem with this argument is that it is not clear that Premise 1 or Premise 2 are as defensible as they may initially appear. First, Premise 1 is not altogether convincing, given that GRS is a clinically indicated procedure supported by medical evidence and experts to treat a recognized medical condition (APA 2013; WPATH 2011). It may involve cosmetic procedures on one level, but it is clearly not *solely* a cosmetic procedure. The objection therefore sets up a false dichotomy between clinical and cosmetic procedures. Second, even if Premise 1 is granted, it is not clear that Premise 2 can be defended. Some cosmetic procedures may be medically warranted for the attainment of an adequate state of mental and physical health, thus falling under the purview of the healthcare system, and thereby refuting Premise 2. Many public health systems, for example, fully fund breast reconstruction surgery for women who have undergone a mastectomy. This is a cosmetic procedure performed on the grounds that it will improve the patient’s mental well-being. Gender reassignment surgery, even if it involves cosmetic procedures, is done for this same reason. Cosmetic and clinical procedures, therefore, often intersect. Gender reassignment surgery is one such circumstance, given its rationale for promoting the mental and physical health of those with diagnosed GD.

The second objection is based on health resource scarcity and the opportunity costs of funding GRS. Information I obtained from the United Kingdom National Health Service (NHS) via a Freedom of Information Request identifies the average cost of one male-to-female GRS at £10,369 (NHS, e-mail message to author, May 22, 2018).^2^ A GRS procedure for a female-to-male, which is far more complex, is an average of £31,780 (NHS, e-mail message to author, May 22, 2018). The majority of GRS performed are for male-to-female assignment, with a total cost to the NHS of £3,525,460 in the financial year of 2016/2017 (NHS, e-mail message to author, May 22, 2018). Using these funds for GRS, it is argued, means unjustifiably depriving other patients of other essential healthcare.

The resource scarcity argument is not convincing. First, GRS can itself be life-saving, and therefore analogous in this way to other essential healthcare services such as intensive care and emergency surgeries that cost more than a single GRS procedure. Without GRS, statistics suggest up to 32 per cent of transgender persons will attempt to commit suicide (Clements-Nolle, Marx, and Katz 2006; Grossman and D’Augelli 2011; Reisner et al. 2016). In purely economic terms, the cost of one death from suicide is identified by some sources at £1.7 million (NHS, 2017a). It may well transpire, therefore, that a cost–benefit or cost–utility analysis would support funding GRS based on the benefits of saving lives, reducing the economic burden on mental health services, and losing fewer years of productive life to suicide.

Second, as far as medical procedures cost the NHS, this is fairly high, though it is comparable to other procedures which are routinely funded, highlighting the issue of consistency. For example, a lung transplant operation costs the NHS £40,076.32 per patient in the financial year 2016/2017 (NHS 2017b). A case of complex tuberculosis costs the NHS £21,598.34 (NHS 2017b). Treatment in an intensive care bed costs £1,932 per night, with a significant portion of patients requiring multiple days of care (NHS 2013, 2017c). Gender reassignment surgery fits within these parameters, given its life-saving and economic benefit, and so consistency demands that we either include it as part of the schedule of publicly funded procedures or identify a morally relevant difference. No such morally relevant difference stands up to critical scrutiny, as I shall later demonstrate.

The third objection to GRS is a slippery slope argument, claiming that if we fund GRS it will lead inexorably to the funding of numerous other procedures. For example, we may have to fund surgery for people who demand rhinoplasty. This objection can be responded to, again, through the principles of consistency. I am willing to accept the implications of this objection if, and only if, the rhinoplasty-seeking person experiences the same adverse health effects as the GD-sufferer. If rhinoplasty will prevent a severely anxious and insecure person from committing suicide, then it seems prima facie justified to publicly fund the procedure. However, the standards required to even be a candidate for GRS are very stringent, and similar standards should apply to the hypothetical life-saving rhinoplasty procedure. The patient must have a genuine and identifiable risk of self-harm and have made an autonomous request, there must be no other viable alternative treatments, and rhinoplasty should have been subjected to the two-level funding evaluation process I shall outline.

A related strand is around race appearance alteration surgery, for example, whether funding GRS means the state would also need to fund a dark-skinned person wanting to make her skin fairer on the basis of the mental distress she feels by being dark-skinned. Unlike GRS, race-based surgery may have morally important third-party effects, such as implicitly making others with dark-skin feel devalued or increasing racial stigma. Changing social attitudes is also the preferred approach since the insecurity stems almost purely from racism in society. While transgender people may be stigmatized in society, the primary effect of GD is the internal turmoil experienced independently of society’s discriminatory attitudes. Even if we removed transgender discrimination in society altogether, the GRS-seeking person would still suffer from the internal psychological distress of not belonging in their physical sex (APA 2013; WPATH 2011). The same does not seem to apply for racism. If we removed racist attitudes from society altogether, it is not clear that the dark-skinned person would continue to experience any distress from their skin colour.^3^

## The Constructive Argument for GRS

Having shown that the common objections against publicly funding GRS do not succeed, I now turn to a constructive argument in favour of such a policy. My constructive argument is to develop a two-level account with which to justify the public funding of GRS. This approach can also serve as a general framework for evaluating other issues of distributive justice in healthcare and is, in fact, likely already used in various jurisdictions around the world in some form or another. The first level of evaluating whether to fund GRS is to first ascertain whether the condition it intends to treat (i.e. GD) fits the criteria of a health issue and, if so, would the treatment (i.e. GRS) enable the person to improve their health. The second level of evaluation is to consider other morally relevant factors, such as opportunity costs of funding the treatment, third party effects, availability of qualified personnel, existence of alternatives, relative utility, and its impact on justice and health equity. The first-level requirement, namely that GD fits the definition of a health issue and that GRS improves health, is therefore a necessary but not sufficient condition for funding. The second level determines ultimately whether or not to fund GRS, given that the first level evaluation has been satisfied, based upon a series of further ethical considerations.

### The First Level of Evaluation

The first stage of the constructive argument is to determine whether or not GRS is a clinically-indicated procedure for a medical condition, based on some definition of health. The definition of health we adopt has profound implications for the two-level approach, since it is the definition that primarily determines whether or not GRS should even be advanced to the second-level for consideration of public funding. At the same time, the definition of health we adopt has implications not only for GRS but for other health conditions more widely. Whatever definition we espouse, we must therefore be prepared to accept its implications and the demands of consistency.

Consider, for example, the World Health Organization’s (WHO) definition of health: “Health is a state of complete physical, mental and social well-being and not merely the absence of disease or infirmity” (WHO 1948, 1–2). If a health system is given the duty of promoting health, as indeed is generally the case, then it follows that its responsibilities under a WHO definition will be very broad indeed. On the other hand, Daniel Callahan rejects the WHO concept of health in favour of a purely physical account, where health is merely a state of physical well-being (Callahan 1973). One implication of Callahan’s account is that mental health conditions would not fall within the purview of health, and so a health system would have no obligations to provide any mental health services.

Both the WHO and Callahan accounts have implications that few of us would likely be willing to accept. With the WHO account, it may transpire that the health system ought to fund or provide a great number of services on the basis that it would promote a person’s complete physical, mental, and social well-being. For example, it may have to provide lonely rural farmers with sex workers to satisfy their social well-being and buy car fanatics Ferraris to promote *complete* mental well-being. With Callahan’s account of health, the state has no duty to provide care for those suffering from severe clinical depression, hallucinations, post-traumatic stress disorder, or any other mental illness regardless of its impact. I suspect few of us would be willing to accept the implications of either of these accounts.

Accordingly, I will present a basic definition of health that is pragmatic, likely to be widely accepted, has plausible implications, and is already in use by most healthcare systems. Space constraints will not allow me to defend or develop this account in any detail, but it will be sufficient to support my present argument. I propose that health is a state of physical and mental well-being. To be healthy is to be in an *adequate* but not necessarily complete nor perfect state of physical and mental health. My basic definition is distinct from Callahan’s account, though not drastically so. The definition does not expand the concept of health to include social and spiritual well-being and can therefore accommodate Callahan’s concerns about the “tyranny of health” where nearly every negative experience ends up being subsumed within the domain of health. The definition also does not give health a utopic or unrealistic goal of *complete* well-being, unlike the WHO’s definition, which Callahan is rightly critical of. However, my account can capture the problem of mental illness as a domain of health, which accords with most clinicians’ and laypeople’s considered judgements about health. It is also how most health systems and funders today view the nature of health and the role of clinicians.

The basic definition of health I propose is sufficientarian rather than “perfectionist” in nature; it strives to reach a certain threshold rather than some absolute standard. The state has no obligation to promote *complete* health in all its citizens. A further clarification should also be made. A condition that fits my basic definition of health does not necessarily mean that the state ought therefore to publicly fund the associated treatments. The role of the definition of health is to clarify and delineate which conditions fit within the purview of the healthcare system. There may be other morally relevant considerations, such as opportunity costs, third party effects, availability of qualified personnel, and existence of reliable clinical evidence. However, these considerations should be addressed in the second-level evaluation after understanding the health condition itself. In general, a condition that falls within the purview of the definition of health should at least be given *consideration* for public funding.

A consistency or derivative argument for the funding of GRS can then be advanced on the basis of this definition of health. First, given the profound effects of GD on mental and physical health, in accordance with our definition, it is a health issue that falls under the purview of the health system. Second, given that we fund a raft of other analogous health conditions on the basis of clinical necessity, for example, depression, anxiety, and other conditions which affect a person’s mental health, consistency demands that we also fund GRS unless we can highlight the presence of morally relevant differences. This argument can, of course, be rebutted by using consistency to argue that we should not fund any of the above treatments for depression or anxiety. However, this would diverge with most reasonable persons’ considered judgement that diagnosable mental health conditions should generally fall within the purview of health. The argument could also be rebutted on the basis of faulty analogy, either on the grounds that GD is not analogous to other mental health conditions or that the treatment for those conditions is not analogous to GRS. This argument, however, is not convincing. The *conditions* are analogous in that they fit our basic definition of health and are diagnosable conditions with a set of accepted guidelines (APA 2013). The *treatments* are analogous in that they are clinically necessary and are based on an attempt to enable a person to improve towards or reach an adequate state of physical and mental health (WPATH 2011).

The claim I am advancing, then, is that what matters for our first level of evaluation is not the specific condition nor the treatment itself, but rather the *effect* of the condition on the person’s state of physical or mental health and the ability for the treatment to help the person attain an adequate state of physical or mental health. This approach means the first-level evaluation is *condition-blind*. It does not matter what specific condition it is as long as it fits our definition of health. Gender dysphoria passes the criteria required in the first-level analysis. Gender dysphoria is a recognized and diagnosable condition, which affects a person’s health per our definition. Gender reassignment surgery, as a course of treatment, is supported by the clinical evidence and is effective for restoring a person to the threshold of physical and mental health or at least greatly improving the transgender person’s health (Wierck, Caenege, and Elaut 2011; WPATH 2011).

### The Second Level of Evaluation

Once the first-level evaluation is completed, namely that the condition (GD) be one that fits our definition of health, we turn to the second-level evaluation to analyse other relevant factors regarding public funding. A number of relevant considerations should be taken into account. One important consideration for publicly funding GRS is the wider distributive justice impact as a result of using scarce health resources. These may include considerations of efficiency, relative utility, and opportunity costs. As already pointed out in my rejoinder to the cost-effectiveness objection to funding GRS, the claims of the critics do not stack up empirically. On the face of it, the economic impact of publicly funding GRS seems favourable (NHS 2017a, 2017b).

Opportunity costs are important considerations in any issue involving health resource allocation, given our finite health budget (Bognar and Hirose 2014; Daniels 2008). The £10,369 to fund one male-to-female GRS could be used for an alternative need, such as funding a certain number of immunizations or a health promotion programme. There must therefore be strong grounds for funding GRS over another procedure. The case for publicly funding GRS is strong, given its potential to be a life-saving procedure and provide immense benefit to the GD patient (APA 2013; WPATH 2011).

Identifying and ethically reasoning about opportunity costs is complex, however, as we cannot be certain that cutting funding from one area will mean it going into another area of essential health need. The £10,369 could, for example, be used to install a new car park for the hospital manager or fund a hospital corporate function instead. Opportunity cost is an important consideration to acknowledge as a general principle of distributive justice in healthcare, but it cannot be the *sole* justification for declining funding unless the treatment is exceedingly expensive such that it would very clearly deprive other patients’ access to an even more important health service. It is not clear that GRS fits this criterion, and so we may not rely *solely* upon its opportunity costs to deny public funding.

Considerations of justice and health equity are morally relevant in deciding to fund GRS. If a particular procedure has especially high benefits for a marginalized or disadvantaged group, we may have extra grounds for supporting it. This could be defended on a number of different grounds including prioritarian distributive justice, whereby we ought to morally give priority to the worst-off; on utilitarian grounds, whereby the principle of diminishing marginal utility posits that the gain in utility is greater for those who are worse off; or on Rawlsian maximin reasoning (Parfit 1997; Rawls 1999). Transgender persons remain one of the most discriminated-against people in society, as well as experiencing poorer physical and mental health than their non-transgender counterparts (Clements-Nolle, Marx, and Katz 2006; Grossman and D’Augelli 2011; Reisner et al. 2016). Gender reassignment surgery improves the mental and physical health of a disadvantaged group, and we may therefore have an increased obligation to publicly fund the treatment on prioritarian, Rawlsian, or utilitarian grounds.

Other considerations at the second-level include the availability of qualified medical and support personnel and the availability of viable alternative treatments. Most developed countries have qualified GRS surgeons, often qualified as plastic surgeons. In the event that no qualified medical personnel are available to perform the procedure, the primary obligation becomes recruiting a workforce that is able to perform GRS. It is not an appropriate response to refuse to publicly fund GRS solely on the basis of the state of the workforce, in the same way that if no qualified clinicians are available to treat schizophrenia, the answer is to recruit such personnel rather than using it as a reason to not treat schizophrenics. As for the existence of viable alternative treatments, WPATH does not actually recommend GRS as a first-line course of treatment for those with GD. In fact, there are strict sets of guidelines for clinicians to follow (WPATH 2011). Gender reassignment surgery is therefore the appropriate treatment for certain people, given that there are no viable alternative treatments available for their GD.

One consideration that may worry policymakers is around consumer behaviour and increased demand for GRS services if it becomes fully funded. A number of responses can be offered to address this concern. First, GD is a recognized condition with diagnostic criteria in the DSM and strict treatment protocols outlined in the WPATH document (APA 2013; WPATH 2011). This fact alone limits the number of those who can make a legitimate claim on the healthcare system to fund their GRS procedure. Second, if the number of people seeking GRS increases as a result of public funding, this will likely be due to more people being able to access a service they needed all along. In such cases, existing clinical need is the driving factor. It is unlikely that people will suddenly “decide” they want to change their gender identity simply because the state now subsidizes GRS. Even if people make decisions on a whim, the criteria in the DSM and WPATH guidelines can respond by declining GRS as an appropriate avenue of treatment. Considerations about inducing demand therefore do not withstand critical scrutiny, and the constructive argument in favour of publicly funding GRS is not affected.

## GRS and Moralistic Bias

The constructive argument in favour of public funding GRS, then, can be summarized as follows: First, GD is a recognized clinical condition with diagnosable criteria. It passes the first stage of evaluation, namely that the condition we are treating be one that falls within the purview of the health per our sufficientarian definition (APA 2013; WPATH 2011). Second, GRS is an effective and evidence-based procedure with clear guidelines, and one that is clinically indicated for the treatment of GD (WPATH 2011). Third, considerations of other morally relevant factors do not damage the constructive case to publicly fund GRS. Gender reassignment surgery is cost-effective, and the opportunity costs are worth incurring given its strong potential to be a life-saving procedure (Clements-Nolle, Marx, and Katz 2006; Grossman and D’Augelli 2011; NHS 2017a; WPATH 2011). There are strong justice-based considerations grounded in prioritarian, utilitarian, or Rawlsian theory to fund GRS, given that transgender people are one of the most disadvantaged groups in society (Reisner et al. 2016). Qualified medical personnel are available to carry out the procedure, and there are no other alternative treatments in the subset of GD patients for whom GRS is clinically indicated (APA 2013; WPATH, 2011).

The constructive argument I have presented in favour of publicly funding GRS may strike some as surprisingly straightforward. However, the fact that people place such a high burden on having to justify an evidence-based, potentially life-saving medical procedure for a medically recognized condition shows that other biases may be at play. These “moralistic biases” refer to existing views and intuitions people may have about GRS and transgender and gender identity issues in general. If there were a pill that could alleviate a person’s severe psychological distress and prevent them from committing suicide at a one-off cost of £10,369, I suspect the burden of justification to fund it would be significantly less than what is demanded for GRS. The fact that we do not place such a high burden of justification for even more expensive life-saving procedures such as transplants, intensive care, and emergency department treatment shows that there are other intuitions at play in the GRS funding debate.

One of these other intuitions could be an implicit bias against altering the human body in any way (NHS 2018). However, altering the human body is often an essential part of medical procedures—appendectomy for the treatment of appendicitis, amputation of a limb with gangrene, or even breast reduction surgery to alleviate weight for those with back problems. These critics would very likely not object to these procedures. The intuition, then, cannot merely be about objecting to altering the body. The biases people have against GRS is probably that they do not see it as a real, medical condition that warrants clinical intervention. Given the large body of medical and scientific evidence about GD and GRS, the burden of proof now rests with those who are attempting to oppose the clinical consensus (APA 2013; Ministry of Health 2012; NHS 2017a, 2018; WPATH, 2011). In the absence of a cogent rebuttal of the clinical consensus, we should treat GRS as merely another clinical procedure for a recognized condition.

Another subset of those who oppose the public funding of GRS could be those influenced by conservative or religious views about the rights of transgender people (NHS 2018; Schwartz and Lindley 2009). In a secular, liberal state, this would arguably be problematic (Raz 1986). Decisions about which medical procedures to fund should be informed by clinical evidence, economic analysis, and sound ethical reasoning. If we allow religious views to dictate which clinical procedures to provide, we may find a host of services being opposed, including contraception and sexual health services. Regardless, my constructive argument does not necessarily depend on subscribing to the transgender rights movement. The argument is driven primarily by the notions of clinical necessity, as well as reasoning in an ethically consistent manner given that we fund other analogous life-saving procedures.

Not all opponents of publicly funding GRS are influenced by so-called moralistic biases. The vast majority of people who oppose GRS, I suspect, are simply unaware of the facts surrounding GD and GRS. This is not necessarily through any fault of their own, as the issue is seldom discussed in social or political circles. The lack of awareness of the empirical evidence leads critics of GRS to resort to knee-jerk intuitions, often informed through biases, social attitudes, the media, and prevailing norms. However, once we acknowledge that GD is a recognized clinical condition and that GRS is a cost-effective evidence-based surgical procedure to treat it, it becomes very difficult to continue opposing public funding.

## Conclusion

This paper has argued that the state should publicly fund GRS. First, I have argued that initial objections to the state funding GRS do not withstand critical scrutiny. Second, I have gone on to propose a constructive argument, based on the principles of clinical necessity, cost-effectiveness, justice, and ethical consistency. Given that the procedure is analogous to numerous other cost-effective, evidence-based, life-saving procedures we fund routinely, there is a strong argument for publicly funding GRS. Third, I considered a number of further important factors and objections. None of the objections against publicly funding GRS hold, and several considerations lend further support to my constructive case. Once we overcome our initial biases and moralistic intuitions about GD and GRS, and instead treat it as we would any other condition and medical procedure, the positive case for publicly funding GRS becomes very hard to deny.

## Footnotes

1 Data from Freedom of Information Request. Received from the NHS via email on May 22, 2018.

2 Information obtained through a Freedom of Information Request. Information received 22 May 2018

3 In our non-ideal real world, I will deliberately leave open the question of whether such race-alteration surgeries should be funded if the dark-skinned person is at very serious risk of suicide, as the lesser of two evils.
